# Supplementary figures and images for: Systems-level analyses dissociate genetic regulators of reactive oxygen species and energy production
Source: bioRxiv. 2023 Oct 18:2023.10.14.562276. Preprint. [Version 1] doi: 10.1101/2023.10.14.562276 (PMC10614765; doi:10.1101/2023.10.14.562276)

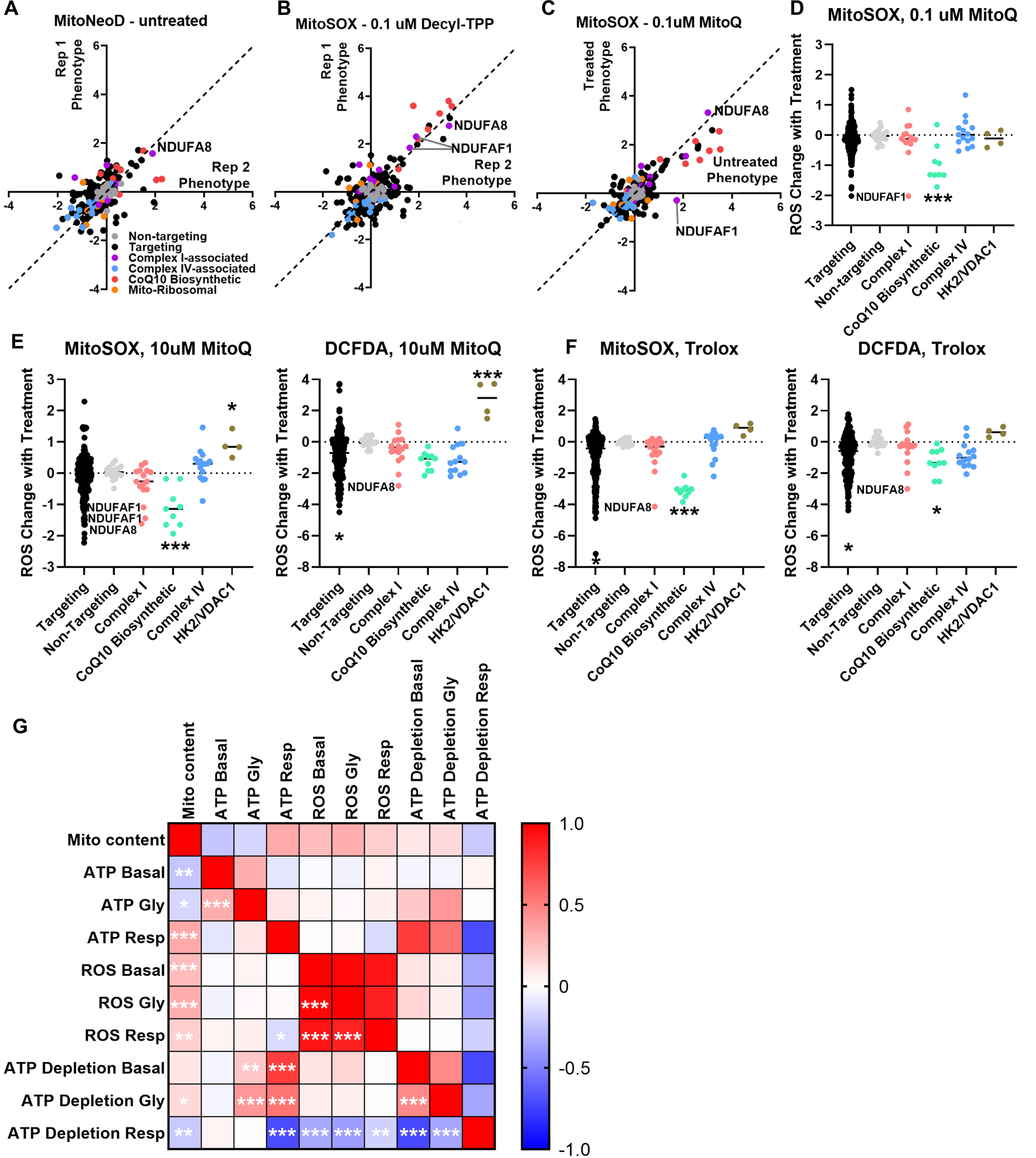

Supplement: Supplement 3 — Fig. S1. Antioxidant effect on ROS levels depends on deficient pathway. A) ROS phenotypes of K562 cells expressing a mini-library of CRISPRi sgRNA detected with MitoNeoD were similar to those measured with MitoSOX, with some of the same hit genes like NDUFA8, and similar patterns with complex IV-associated gene knockdowns having low ROS. B) Some of these same patterns were observed in cells treated with 0.1 µM Decyl-TPP, the control for MitoQ Treatment. Cells treated with either C,D) low dose of MitoQ (0.1 µM) or E) high dose (10 µM) of MitoQ respond differently depending on the genes knocked down. MitoQ at both doses abrogated the increase in ROS from CRISPRi knockdown of CoQ10 biosynthetic genes. In contrast, 10 µM MitoQ treatment increased mitochondrial and cytosolic ROS following knockdown of HK2 and VDAC1. F) Trolox (1 mM) abrogated the increase in ROS following knockdown of CoQ10 biosynthetic genes. Trolox treatment also decreased ROS levels across all targeting CRISPRi sgRNA in aggregate. n = 2 replicates. G) Pearson r correlation matrix of mitochondrial content, ATP level and ATP depletion phenotypes in basal, respiration-only, and glycolysis-only conditions collected previously from n = 2 replicates(13), along with ROS phenotypes in the same conditions in cells expressing CRISPRi knockdown libraries. Across ATP level phenotypes, there was only significant correlation between basal and glycolysis-only conditions. In contrast, ROS levels correlated between all substrate conditions. *p < 0.05, **p < 0.01, ***p < 0.001 by one-way ANOVA with Dunnett’s multiple comparisons test (A,B) and (C) by Pearson correlation test. [file media-3.pdf]

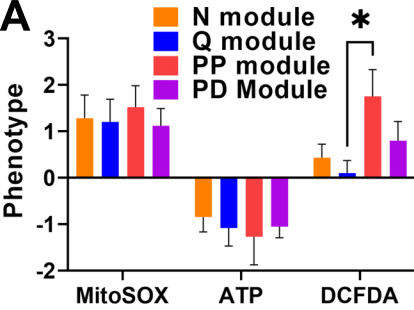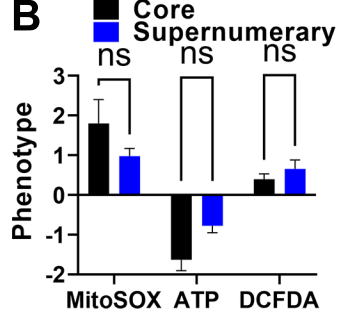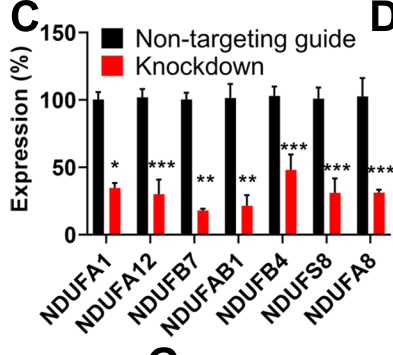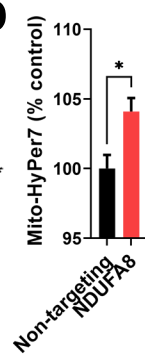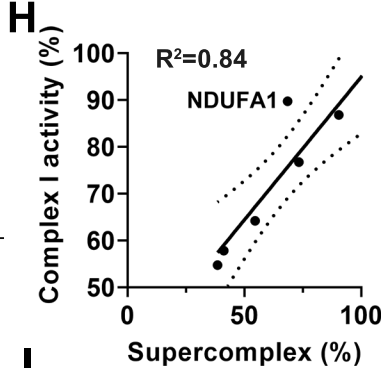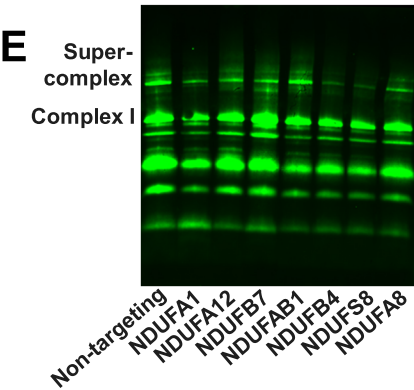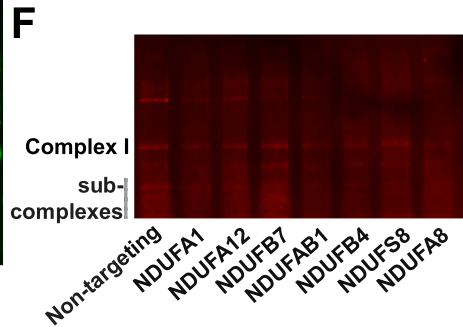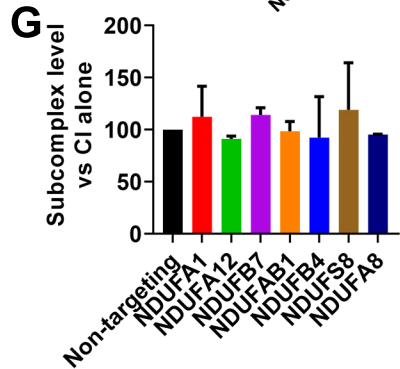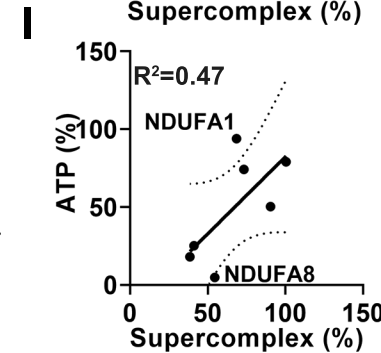

Supplement: Supplement 4 — Fig. S2. CRISPRi knockdown of complex I subunits decreases supercomplex levels. A) Knockdown of subunits within functional modules of complex I did not significantly affect either mitochondrial ROS or ATP. Knockdown of subunits within the Q module and PP module significantly differed in their effects on cytosolic ROS. B) Effect of knockdown of core conserved subunits did not significantly differ from the effect of knockdown of non-core or supernumerary subunits on mitochondrial ROS, cytosolic ROS, or ATP. Data compiled from n = 2 experiments. ATP phenotype data previously collected in Bennett et al (13). C) RT-qPCR of K562 lines expressing CRISPRi sgRNA knocking down individual complex I subunits. n = 2–10 replicates per cell line. D) Knockdown of NDUFA8 in K562 cells increases mitochondrial ROS, as measured by mitochondrial matrix-targeted HyPer7. Data compiled from n=2 experiments. E) Additional representative blue-native PAGE gel replicate, loaded with isolated mitochondria for each of seven cell lines expressing CRISPRi knockdown of complex I subunits and a non-targeting control, and stained with total OXPHOS human western blot antibody cocktail. F) Representative blue-native PAGE gel, loaded with isolated mitochondria for each of seven cell lines expressing CRISPRi knockdown of complex I subunits and a non-targeting control, and stained with NDUFS4-targeting antibody to identify NDUFS4-containing sub-complexes at molecular weights less than fully assembled complex I. G) There were no significant differences in subcomplex levels versus non-targeting controls. n = 2 blue-native PAGE gels Supercomplex levels correlate with complex I activity (H) and ATP levels (I). NDUFA1 knockdown has proportionally higher complex I activity and ATP than other subunits, given its level of supercomplex, indicating that its knockdown may preserve a supercomplex species of higher energetic productivity. Trendline shows linear regression across the complex I knockdown cell lines, and [file media-4.pdf]
